# Correction to “Hypoxia promotes EV secretion by impairing lysosomal homeostasis in HNSCC through negative regulation of ATP6V1A by HIF‐1α”

**DOI:** 10.1002/jev2.12330

**Published:** 2023-05-27

Wang, X., Wu, R., Zhai, P., Liu, Z., Xia, R., Zhang, Z., Qin, X., Li, C., Chen, W., Li, J., & Zhang, J. (2023). Hypoxia promotes EV secretion by impairing lysosomal homeostasis in HNSCC through negative regulation of ATP6V1A by HIF‐1α. *Journal of Extracellular Vesicles*, 12, e12310. https://doi.org/10.1002/jev2.12310

## 1 IN THE ORIGINAL ARTICLE, TWO ADDITIONAL CORRESPONDING AUTHORS WERE NOT INCLUDED. THE COMPLETE LIST OF CORRESPONDING AUTHORS IS AS FOLLOWS

Jianjun Zhang, Ninth People's Hospital, Shanghai Jiao Tong University School of Medicine, 639 Zhizaoju Road, Shanghai 200011, China. Email: zjjbio@sjtu.edu.cn

Jiang Li, Ninth People's Hospital, Shanghai Jiao Tong University School of Medicine, 639 Zhizaoju Road, Shanghai 200011, China. Email: LIJ1042@sh9hospital.org.cn

Wantao Chen, Ninth People's Hospital, Shanghai Jiao Tong University School of Medicine, 639 Zhizaoju Road, Shanghai 200011, China. Email: chenwantao196323@sjtu.edu.cn

We apologize for this error.

